# Microrheology with Optical Tweezers: Measuring the relative viscosity of solutions ‘*at a glance*'

**DOI:** 10.1038/srep08831

**Published:** 2015-03-06

**Authors:** Manlio Tassieri, Francesco Del Giudice, Emma J. Robertson, Neena Jain, Bettina Fries, Rab Wilson, Andrew Glidle, Francesco Greco, Paolo Antonio Netti, Pier Luca Maffettone, Tihana Bicanic, Jonathan M. Cooper

**Affiliations:** 1 Division of Biomedical Engineering, School of Engineering, University of Glasgow, Glasgow G12 8LT, UK; 2 Center for Advanced Biomaterials for Health Care @CRIB, IIT, P.le Tecchio 80, 80125 Naples, Italy; 3 Department of Infection and Immunity, St George's University of London, London SW17 0RS, UK; 4 Department of Medicine, Albert Einstein College of Medicine, Bronx NY, USA; 5 Istituto di Ricerche sulla Combustione, IRC-CNR, P.le Tecchio 80, 80125 Naples, Italy; 6 Dipartimento di Ingegneria Chimica, dei Materiali e della Produzione Industriale, , Universitá di Napoli Federico II, P.le Tecchio 80, 80125 Naples, Italy

## Abstract

We present a straightforward method for measuring the relative viscosity of fluids *via* a simple graphical analysis of the normalised position autocorrelation function of an optically trapped bead, without the need of embarking on laborious calculations. The advantages of the proposed microrheology method are evident when it is adopted for measurements of materials whose availability is limited, such as those involved in biological studies. The method has been validated by direct comparison with conventional bulk rheology methods, and has been applied both to characterise synthetic linear polyelectrolytes solutions and to study biomedical samples.

The pioneering studies of Albert Einstein^1^ introduced one of the most important parameters in the field of solution rheology: the *relative viscosity* (*η_r_*), defined as the ratio of the solution viscosity (*η*) to that of the solvent (*η_s_*). Einstein indeed derived an expression for the relative viscosity of a suspension of hard spheres at low volume fractions (i.e. 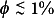): *η_r_* = 1 + 2.5*ϕ*. This was the spark that led to a myriad of studies^2^,^3^,^4^,^5^ seeking to find the *yet undefined* laws governing the rheology of highly concentrated (i.e. for 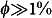) suspensions.

With the advent of polymer physics, scientists established i) that, for very dilute polymer solutions, the viscosity increases above the solvent viscosity linearly with the polymer mass concentration, *c*, and ii) that the effective ‘virial expansion' for relative viscosity is: *η_r_* = 1 + [*η*]*c* + *κ_H_*[*η*]^2^*c*^2^ +..., where [*η*] is the so-called intrinsic viscosity and *k_H_* is the Huggins coefficient^6^. The intrinsic viscosity can be seen as the linear extrapolation to zero concentration of the *reduced viscosity η_red_* ≡ (*η_r_* − 1)/*c*, when this is plotted against mass concentration.

The ability to determine the intrinsic viscosity of polymers from rheological measurements became of interest to a broad scientific community when it was found that [*η*] is simply related to the polymer molecular weight (*M*) by means of the Mark–Houwink equation: [*η*] = *KM^α^*, where *K* and *α* are two constants that have been tabulated for a variety of polymers in various solvents^7^,^8^. Over the time, both the concentration dependence of *η_r_* and the resulting [*η*] have been correlated, both theoretically and experimentally, to the size, shape, mass and intermolecular interactions of the solute molecule^6^,^7^,^8^,^9^,^10^,^11^,^12^,^13^, and to the solvent ‘quality'; hence the importance of their knowledge.

Conventionally, there are two popular methods for measuring *η_r_*: the first is based on the use of an Ubbelohde viscometer, which requires the measurement of the liquids' efflux times through a thin capillary of known geometries; the ratio between the measured times of a pair of fluids is simply proportional to their *η_r_*^14^. The second method involves the measurement of the liquids' steady speeds of deformation (i.e. the shear rates) occurring as consequence of a known applied constant stress; the ratio between the measured shear rates provides a measure of the relative viscosity of two fluids, if the stress is kept the same in both the measurements.

Despite their simplicity, both of the above methods require tens of millilitres of sample volume, resulting in being unsuitable for rare or precious materials such as those involved in biological studies^15^,^16^,^17^,^18^,^19^. This emphasises the importance of *new* experimental methods^20^,^21^,^22^ for measuring *η_r_*, as the one we are going to propose here, by means of optical tweezers (OT). Indeed, like other microrheology techniques^23^,^24^,^25^, the presently proposed technique only requires a few microlitres of sample volume per measurement and provides a straightforward and accurate procedure for measuring the relative viscosity of solutions – and therefore the materials's molecular weight *via* their intrinsic viscosity *plus* the Mark–Houwink law. We demonstrate how this result can be achieved by means of a simple analysis of the normalised position autocorrelation function of an optically trapped bead, with the added advantage of avoiding laborious calculations that would involve either Laplace/inverse-Laplace *or* Fourier transformations of discrete time-dependent experimental data^26^,^27^,^28^,^29^,^30^.

In order to validate the proposed method, we have determined the molecular weight of two known polyacrylamides (PAMs) by means of their intrinsic viscosities, which have been extrapolated from relative viscosity measurements, performed with OT, on water-based solutions of the two PAMs at different concentrations, as described hereafter. Moreover, we report measurements of the relative viscosity, over a wide range of concentrations, for water-based solutions of the polysaccharide glucuronoxylomannan (GXM), which is a major constituent of the capsule of the *Cryptococcus neoformans* and a well-characterised virulence factor with immunomodulatory properties^16^,^31^. The proposed method has been validated by direct comparison with conventional bulk rheology techniques for all three the above systems.

## Results and Discussion

**Figure 1:**
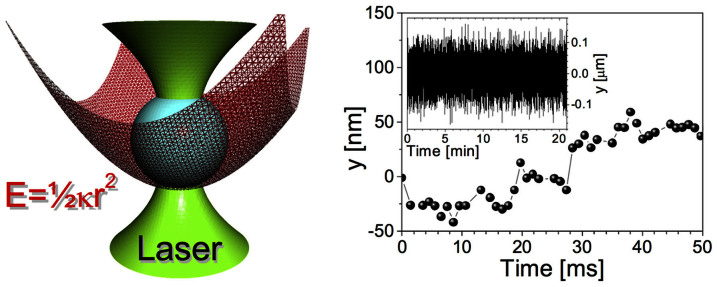
Optical Tweezers and particle trajectory. (Left) A schematic representation of an optically trapped bead within a harmonic potential *E*(*r*), where *κ* and *r* are the trap stiffness and the bead position from the trap centre, respectively. (Right) The *y*-component of the trajectory of an optically trapped bead of 2.5 *μm* radius (*a*) suspended in water over a period of 50 *ms*. The inset shows the same component as before, but over the entire experiment of 22 *min*.

**Figure 2:**
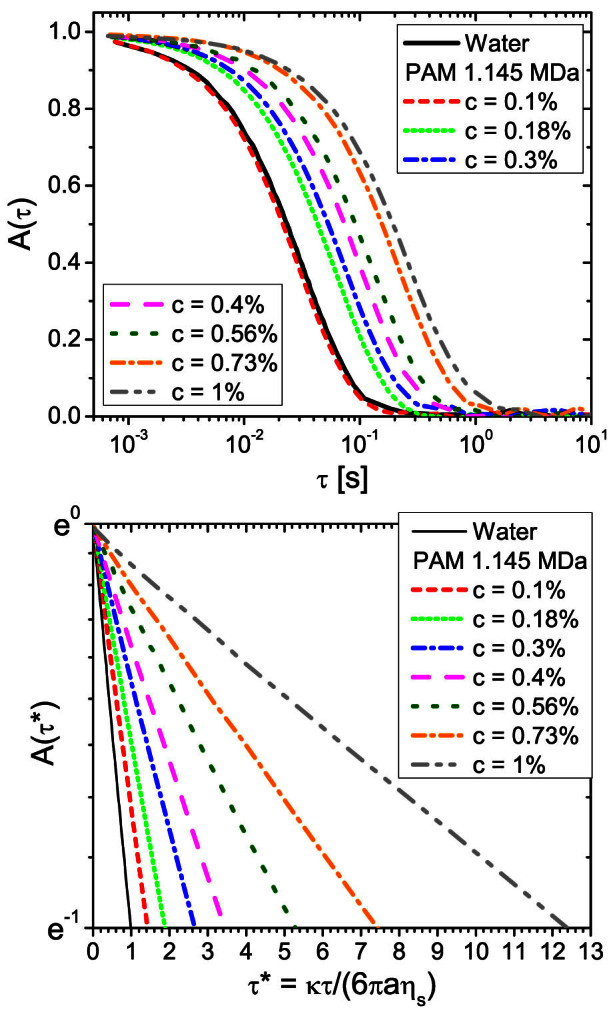
The particle normalised position autocorrelation function. (Top) Linear-Log plot of the normalised position autocorrelation function *A*(*τ*) *vs.* lag-time *τ* of a microsphere suspended in water and water-based solutions of polyacrylamide (*M* = 1.145 *MDa*) at concentrations ranging from 0.1% *w*/*w* to 1% *w*/*w*. (Bottom) Ln-linear plot of the same data as shown above, but drawn *vs.* a dimensionless lag-time τ* = *τκ*/(6*πaη_s_*), where *η_s_* is the solvent viscosity. The ordinate axis has been limited to the region of interest, i.e. *A*(τ*)∈[*e*^−1^,1].

When a micron-sized spherical particle is suspended into a fluid at thermal equilibrium, and is constrained by the stationary harmonic potential generated by OT, the particle's stochastic trajectory (see Fig. 1) is governed by both the thermal fluctuations of the surrounding fluid's molecules and the restoring force exerted by OT. Therefore, a statistical analysis of the bead trajectory has the potential of revealing both the trap stiffness (*κ*) and the fluids' linear viscoelastic properties^30^,^32^,^33^,^34^,^35^,^36^. In the simplest case when a particle of radius *a* is suspended into a Newtonian fluid (i.e. a fluid with constant viscosity *η*), the particle normalised position autocorrelation function (NPAF, see Fig. 2) assumes the form of a single exponential decay^30^:
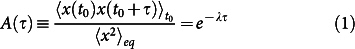
where *λ* = *κ*/(6*πaη*) is the characteristic relaxation rate of the compound system (also known as the “*corner frequency*”^37^). In the NPAF definition, the brackets 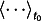 indicate the average taken (by time-translation invariance) over all initial times *t*_0_ within a single trajectory, *τ* is the lag-time (or time interval (*t* − *t*_0_)), *x*(*t*) is the one dimensional particle position from the trap centre (which is assumed to be coincident with the origin of the coordinate system), and 〈*x*^2^〉*_eq_* is the equilibrium variance of the bead position. Notably, for *sufficiently* long measurements (i.e., much longer than *λ*^−1^), 〈*x*^2^〉*_eq_* can in fact be used to calibrate the trap stiffness^30^, by appealing to the Principle of Equipartition of Energy:
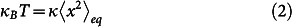
where *k_B_* is the Boltzmann's constant and *T* is the absolute temperature. Notice that, such calibration method is independent from the rheological nature of the fluid under investigation, i.e. from its Newtonian/non-Newtonian character. However, when changing the suspending fluid (e.g., by changing concentration) or the laser properties (e.g., the laser power), the calibration procedure to determine *κ* must of course be repeated, because variations of the experimental conditions will affect the measured variance 〈*x*^2^〉*_eq_*, hence the stiffness *κ*.

From equation (1), with a simple change of variables, it is possible to write:
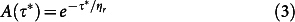
where τ* = *τκ*/(6*πaη_s_*) is a *dimensionless* lag-time containing the pure solvent Newtonian viscosity *η_s_*. (Hereafter the solvent is simply water, with *η_s_* = 0.896 *mPa* · *s*.) It follows that, in an *A*(τ*) *vs* τ* plot, by drawing a horizontal line starting from the ordinate *e*^−1^, the abscissa of its intercept with *A*(τ*) provides a *direct reading* of the solution relative viscosity *η_r_*. Notably, in the case of pure solvent (water), the abscissa of the intercept of *e*^−1^ with *A*(τ*) is 1. In Figure 2, we show the results of such ‘graphical procedure', for water-based solutions of polyacrylamide (*M* = 1.145 *MDa*) at concentrations ranging from 0% *w*/*w* (pure water) to 1% *w*/*w*, hence also well beyond the dilute limit.

**Figure 3:**
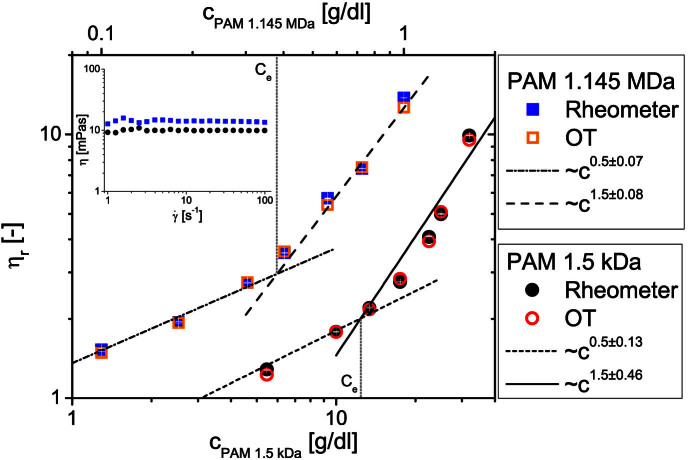
Comparison between micro- and bulk-rheology measurements of the relative viscosity. The relative viscosity *vs.* concentration of water-based solutions of two polyacrylamides having molecular weights of 1.5 *kDa* (bottom axis) and 1.145 *MDa* (top axis). The filled and the open symbols refer to bulk- and micro-rheology measurements of *η_r_*, respectively. The white symbols indicate the error bars of the bulk-rheology measurements. The lines are best fits of the power laws. The entanglement concentrations are indicated by the dot-lines for both the PAMs: i.e., *c_e_* = 12.4 *g*/*dl* and *c_e_* = 0.38 *g*/*dl*, respectively. The inset shows two examples of shear viscosity measurements performed on two solutions having the highest concentrations of PAMs explored in this work, i.e. *c* = 32 *g*/*dl* and *c* = 1 *g*/*dl*, respectively.

In general, polymer solutions as those employed in this work are non-Newtonian, especially at relatively high concentrations where the viscosity may not be constant, particularly at high frequencies (or high shear rates). It follows that equation (1) may not be valid for those solutions, at least not for all concentrations. In dilute conditions, however, at relatively low polymer concentrations, most of solutions tend to show a Newtonian behaviour over a wide range of shear rates (e.g. see inset of figure 3), and this is especially so towards vanishingly small values of concentration, which *coincidentally* are the same conditions required for measuring [*η*]. Hence, the applicability of equations (1) and (3) for measuring *η_r_*, and therefore [*η*], is confirmed in those conditions.

When considering more concentrated solutions, we can argue as follows. It is always possible to write the NPAF in the general form:
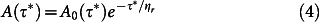
The above described graphical procedure will therefore stay valid in general, i.e. for whatever solution concentration, if the following two conditions are met: i) 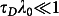, where *τ_D_* is the fluid's longest relaxation time and *λ*_0_ = *κ*/(6*πaη*_0_) is the system corner frequency evaluated with the solution zero-shear viscosity *η*_0_; ii) 
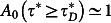
, where 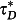 is a dimensionless time equals to *τ_D_*/(6*πaη_s_*/*κ*). Condition ii) simply states that the *A*(τ*) function decays exponentially beyond 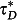, and, more importantly, that its ‘initial decay', i.e. below 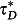, is *small* when compared to its ‘complete decay', up to *A*(−) = 1/*e*. When taken together, therefore, conditions i) and ii) simply state that there exists a *large* range of lag-times 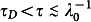, where the decay described by equation 4 is in fact indistinguishable from one mathematically described through equation 3. (Notice that, for 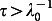 the elastic component of the OT dominates the particle dynamics whatever the nature of the surrounding fluid).

It is clear that, with the two just mentioned conditions fulfilled, the graphical procedure illustrated above will work properly even under non-dilute conditions, which, as a matter of fact, is the situation shown in Figure 2. Of course, in rheological investigations, the fulfilling of conditions i) and ii) above is uncertain prior of performing the measurements, simply because the parameters *τ_D_* and *η*_0_ are yet unknown. As a practical recipe, therefore, we should simply look, when measuring the NPAF, to the linearity of the log-linear plot of *A*(τ*) *vs* τ*; if linearity is there (as it is, e.g., in Figure 2 bottom panel), then *η_r_* can be directly obtained, irrespective of other rheological measurements aimed to ascertain the Newtonian/non-Newtonian nature of the solution under investigation.

**Figure 4:**
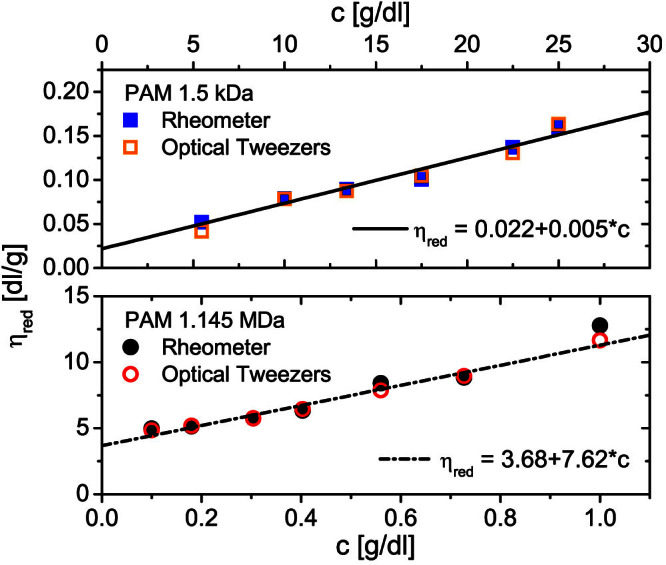
Extrapolation of the intrinsic viscosity. The reduced viscosity *vs.* concentration derived from the data shown in Figure 3. The lines are linear fits of OT data. The linear extrapolation of *η_red_* to zero concentration provides a *reading* of the PAMs' intrinsic viscosities.

In order to validate the proposed method, in Figure 3 we compare the relative viscosity measurements obtained form OT, as described above, with those gained from a conventional stress-controlled rheometer of water-based solutions of two polyacrylamides having molecular weights of 1.5 *kDa* and 1.145 *MDa*. Quantitative agreement between micro- and bulk-rheology is apparent. Notice that, for both the PAMs, the concentration dependence of the relative viscosity obey the theoretical predictions of the concentration scaling-laws for linear polyelectrolytes^11^: i.e., *η_r_* ∝ *c*^0.5^ for the semi-dilute regime (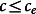) and *η_r_* ∝ *c*^1.5^ for the entangled regime (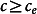). The entanglement concentrations *c_e_* for both the PAMs at the transition between such two regimes can also be clearly identified: i.e., *c_e_* = 12.4 *g*/*dl* and *c_e_* = 0.38 *g*/*dl*, respectively. Notably, the ratio between these two values (i.e., 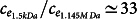) is in good agreement with the theoretical prediction^11^ of *c_e_* ∝ *M*^−0.5^, which in this case results in: 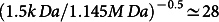. The conclusion, as anticipated above, is that the relative viscosity direct reading (“*at a glance*”) through equation (4) plus conditions i) and ii) is quite reliable, even though our polymer solutions are rather far from their infinite dilution limit. Once the relative viscosities of the solutions are known, it is a simple step to reorganise the data in terms of reduced viscosities *η_red_*, as shown in Figure 4. The linear extrapolation of *η_red_* to zero concentration provides a *reading* of the PAMs' intrinsic viscosities. These, as introduced earlier, are simply related to the polymers molecular weight by means of the Mark–Houwink equation, which for water-based solutions of polyacrylamides can be written as^38^:
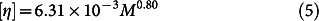

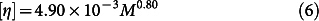
where the above two equations are valid for PAMs with low (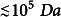) and high (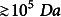) molecular weights, respectively. In Table 1 we report the results obtained by substituting the values of [*η*] derived from Figure 4 in both equations (5) and (6) and compare them with the nominal values of the molecular weights provided by the supplier; the agreement is good, especially when considering the respective range of validity of the two equations.

Having established the efficacy of the new method, we proceed to measure the relative viscosities of an important biological solution, for which conventional rheology is almost prohibited due to the sample volumes availability. Specifically, we have investigated solutions of glucuronoxylomannan (GXM), which is a polysaccharide extracted from clinical isolates of the fungus *Cryptococcus neoformans*. GXM is a major constituent of the capsule of the *Cryptococcus neoformans* and a well-characterised virulence factor with immunomodulatory properties^16^,^31^.

**Figure 5:**
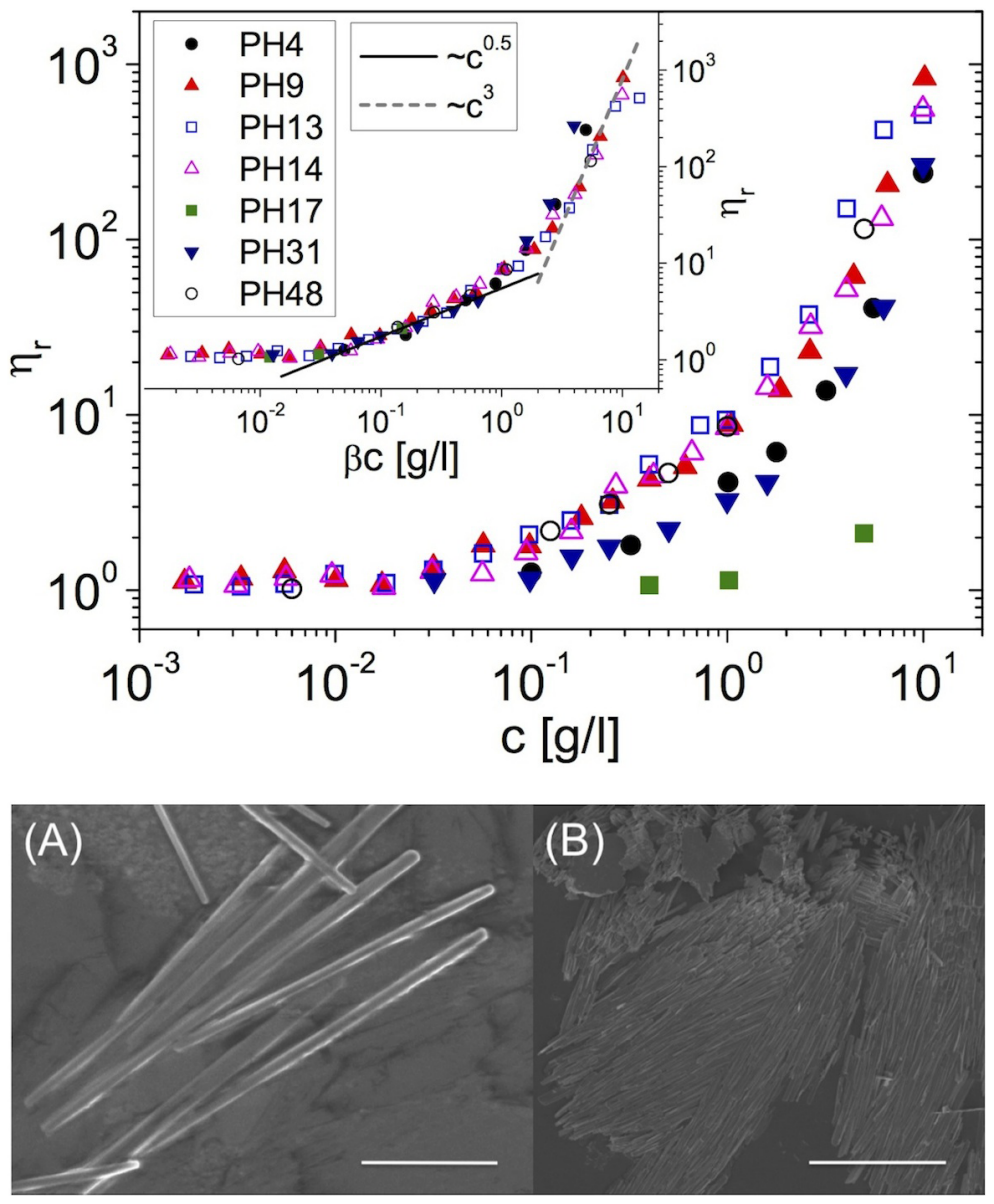
Microrheology of Glucuronoxylomannan solutions. (Tob) The relative viscosity *vs.* concentration of water-based solutions of GXMs isolated from *in vitro* culture of seven clinical *C. neoformans* isolates: PH4, PH9, PH13, PH14, PH17, PH31, PH48. The inset shows the same data, but horizontally shifted by an arbitrary scaling factor *β*. The lines are guides for the power laws. (Bottom) Scanning electron microscope images of rod-shaped GXM crystals obtained from clinical *C. neoformans* isolates PH9 (A) and PH13 (B). The scale bars are 25 *μm* in (A) and 50 *μm* in (B).

Using optical tweezers, we have measured the relative viscosities of purified GXMs dissolved in ultra-pure water, at concentrations ranging from 0.002 *g*/*l* to 10 *g*/*l*, as shown in Figure 5 (top). In particular, the solutions were prepared with GXM exo-polysaccharide isolated from *in vitro* culture of seven clinical *C. neoformans* isolates. From the results shown in Figure 5 (top), together with the master curve shown in its inset (where the same data are scaled horizontally by an arbitrary factor *β*), one can infer the existence of a common physical process governing the concentration scaling-laws of *η_r_* for all the GXM solutions investigated. In this regard, it is interesting to notice the clear existence of two power-laws governing the concentration dependency of *η_r_*, which are typical of rod-like polymer solutions: i.e., *η_r_* ∝ *c*^0.5^ for the semi-dilute regime and *η_r_* ∝ *c*^3^ for the entangled regime^10^. Notably, these results are supported by scanning electron microscope images, which reveal the existence of rod-shaped GXM crystals, as shown for example in Figure 5 (bottom) for two samples obtained from clinical *C. neoformans* isolates PH9 (A) and PH13 (B).

**Figure 6:**
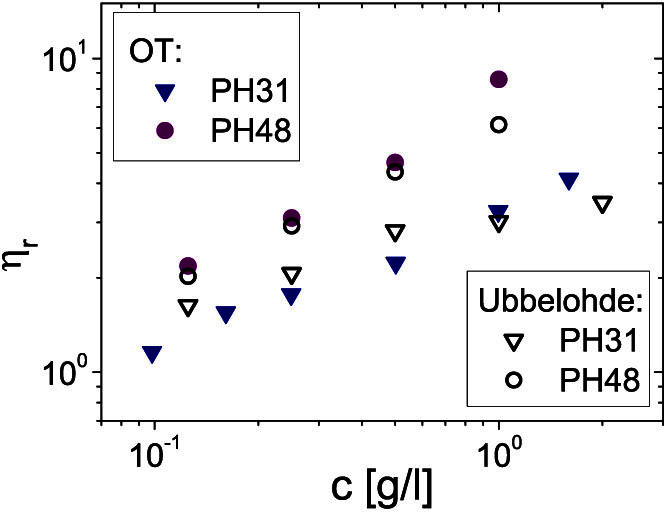
Comparison between Optical Tweezers and Ubbelohde rheological measurements. The relative viscosity *vs.* concentration of two GXM solutions derived from clinical isolates of *Cryptococcus* PH31 and PH48. The graph shows a comparison between measurements of *η_r_* performed with OT (filled symbols) and an Ubbelohde viscometer (open symbols).

Finally, in order to provide further experimental evidence of the validity of the proposed microrheology method for measuring *η_r_*, we compare micro- and bulk-rheology measurements of *η_r_* for two GXM strands derived from clinical isolates of *Cryptococcus* PH31 and PH48, as shown in Figure 6. The close agreement between the results obtained from two distinct techniques (i.e., OT and Ubbelohde) provides additional confidence to the effectiveness of the method.

In conclusion, we have introduced and validated a simple experimental method for measuring the relative viscosity of solutions with optical tweezers, by means of a direct visual inspection (‘*at a glance*') of the particle normalised position autocorrelation function. It should be emphasised that all the rheological results obtained in this paper come from ‘single-run' OT experiments, but are nevertheless very accurate, as shown by the comparison with the results obtained through classical standard techniques.

The advantages of the proposed method rely not only on its simplicity, but also on its microrheology nature (i.e. it requires micro-litres sample volume), which makes it of great interest to all those studies where rare and precious materials are involved, such as biomedical studies.

## Methods

### Optical tweezers

The optical tweezers are built around an inverted microscope, where the same objective lens (100×, 1.3 numerical aperture, oil immersion, Zeiss, Plan-Neouar) is used both to focus the trapping beam and to image the thermal fluctuations of a 5 *μm* diameter silica bead positioned at least 30 *μm* far from the closest wall (i.e. the glass coverslip). Optical trapping is achieved by means of a titanium-sapphire laser with a 5*W* pump (Verdi V5 laser; Coherent Inc.), which provides up to 1*W* at 830 nm. A complementary metal-oxide semiconductor camera (Dalsa Genie HM640 GigE) takes high-speed images of a reduced field of view. These images are processed in real-time at 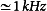 using our own LabVIEW (National Instruments) particle tracking software^39^. All the measurements were performed at a temperature of 25°*C* ± 0.2°*C*.

### Rheological Measurement

Rheological measurements were performed by using a stress-controlled rotational rheometer (Anton-Paar Instrument MCR-302) able to detect torque values down to ~0.1 *μNm*. The rheometer was equipped with a solvent trap to avoid evaporation, and with a cone and plate geometry of 50 *mm* diameter and 1° angle. All the measurements were performed at a constant temperature of 22°*C* ± 0.01°*C*.

### Scanning Electron Microscopy

Scanning electron microscope (SEM) images of the GXMs solutions were taken after drying them at room temperature on glass coverslips, the samples were then sputtered coated with approximately 3 *nm* of AuPd prior to viewing on a Hitachi S3000 scanning electron microscope with a maximum resolution of 3.5 *nm* at 25 *kV*.

### Fluids

Two polyacrylammides (PAMs, from Polysciences Inc.) having nominal molecular weight of *M_w_* = 1, 500 *Da* and of *M_w_* = 1, 145, 000 *Da* were used to prepare aqueous solution at mass concentrations ranging from ≈5% *w*/*w* to ≈40% *w*/*w* and from ≈0.1% *w*/*w* to ≈1% *w*/*w*, respectively. The solutions were stirred at 200 *rpm* for 48 *h* at room temperature.

The protocol for the production and purification of the Glucuronoxylomannan (GXM) from the capsule of the *Cryptococcus neoformans* has been described by Robertson E.J. *et al.*^16^. GXMs solutions were prepared by dissolving purified GXMs in ultra-pure water. The explored concentrations range from 0.002 *g*/*l* to 10 *g*/*l*. The solutions were stirred at 200 *rpm* for 2 *h* at room temperature.

## Figures and Tables

**Table 1:** Comparison between nominal and measured molecular weights of two commercially available polyacrylamides

[*η*] (*dl*/*g*)	Equation –	*M* (*Da*)	*M* (*Da*)
–	*Nominal*	*1,500*	*1,145,000*
0.022 ± 0.008	Equation (5)	**1,506 ± 685**	906, 302 ± 73, 883
3.68 ± 0.24	Equation (6)	2, 066 ± 939	**1,240,000 ± 101,353**

## References

[b1] Einstein A. A new determination of the molecular dimensions (vol 19, pg 289,1906). AdP 34, 591–592 (1911).

[b2] Batchelor G. K. The effect of Brownian motion on the bulk stress in a suspension of spherical particles. J. Fluid Mech. 83, 97–117 (1977).

[b3] Krieger I. M. & Dougherty T. J. A Mechanism for Non-Newtonian Flow in Suspensions of Rigid Spheres. Trans. Soc. Rheol. 3, 137–152 (1959).

[b4] Mueller S., Llewellin E. W. & Mader H. M. The rheology of suspensions of solid particles. Proc. R. Soc. A 466, 1201–1228 (2010).

[b5] Boyer F., Guazzelli E. & Pouliquen O. Unifying Suspension and Granular Rheology. Phys. Rev. Lett. 107 10.1103/PhysRevLett.107.188301 (2011). 22107679

[b6] Ferry J. D. Viscoelastic properties of polymers 3rd edn. (Wiley, 1980).

[b7] Rubinstein M. & Colby R. H. Polymer Physics. (Oxford University Press, USA, 2003).

[b8] Mark J. E. Physical properties of polymers handbook 2nd edn. (Springer, 2007).

[b9] Doi M. & Edwards S. F. Dynamic of rod-like macromolecules in concentrated-solution. 1. J. Chem. Soc. Faraday Trans. II 74, 560–570 (1978).

[b10] Doi M. & Edwards S. F. Dynamic of rod-like macromolecules in concentrated-solution .2. J. Chem. Soc. Faraday Trans. II 74, 918–932 (1978).

[b11] Dobrynin A. V., Colby R. H. & Rubinstein M. Scaling Theory of Polyelectrolyte Solutions. Macromolecules 28, 1859–1871 (1995).

[b12] Di Cola E. *et al.* Persistence length of titin from rabbit skeletal muscles measured with scattering and microrheology techniques. Biophys. J. 88, 4095–4106 (2005). 15792980 10.1529/biophysj.104.054908 PMC1305640

[b13] Di Cola E., Waigh T. A. & Colby R. H. Dynamic light scattering and rheology studies of aqueous solutions of amphiphilic sodium maleate containing copolymers. J. Polym. Sci. Part B Polym. Phys. 45, 774–785 (2007).

[b14] Viswanath D. S., Ghosh T. K., Prasad D. H. L., Dutt N. V. K. & Rani K. Y. Viscosity of liquids: theory, estimation, experiment, and data. (Springer, 2007).

[b15] Frases S. *et al.* The Elastic Properties of the Cryptococcus neoformans Capsule. Biophys. J. 97, 937–945 (2009). 19686640 10.1016/j.bpj.2009.04.043 PMC2726329

[b16] Robertson E. J. *et al.* *Cryptococcus neoformans* Ex Vivo Capsule Size Is Associated With Intracranial Pressure and Host Immune Response in HIV-associated Cryptococcal Meningitis. J. Infect. Dis. 209, 74–82 (2014). 23945372 10.1093/infdis/jit435 PMC3864387

[b17] Watts F. *et al.* Investigating the micro-rheology of the vitreous humor using an optically trapped local probe. J. Opt. 16, 10.1088/2040-8978/16/1/015301 (2014).

[b18] Tassieri M. *et al.* Dynamics of Semiexible Polymer Solutions in the Highly Entangled Regime. Phys. Rev. Lett. 101, 10.1103/PhysRevLett.101.198301 (2008). 19113316

[b19] Tassieri M., Evans R. M. L., Barbu-Tudoran L., Trinick J. &Waigh T. A. The self-assembly, elasticity, and dynamics of cardiac thin filaments. Biophys. J. 94, 2170–2178 (2008). 18065478 10.1529/biophysj.107.116087 PMC2257900

[b20] Grupi A. & Minton A. P. Capillary Viscometer for Fully Automated Measurement of the Concentration and Shear Dependence of the Viscosity of Macromolecular Solutions. Anal. Chem. 84, 10732–10736 (2012). 23130673 10.1021/ac302599j PMC4166550

[b21] Kang Y. J. & Yang S. Integrated microfluidic viscometer equipped with fluid temperature controller for measurement of viscosity in complex fluids. Microfluid Nanofluidics 14, 657–668 (2013).

[b22] Gachelin J. *et al.* Non-Newtonian Viscosity of *Escherichia coli* Suspensions. Phys. Rev. Lett. 110 10.1103/PhysRevLett.110.268103 (2013). 23848926

[b23] MacKintosh F. C. & Schmidt C. F. Microrheology. Curr. Opin. Colloid Interface Sci. 4, 300–307 (1999).

[b24] Waigh T. A. Microrheology of complex fluids. Rep. Prog. Phys. 68, 685–742 (2005). 10.1088/0034-4885/79/7/074601 27245584

[b25] Squires T. M. & Mason T. G. Fluid Mechanics of Microrheology. Annu. Rev. Fluid Mech. 42, 413–438 (2010).

[b26] Mason T. G. & Weitz D. A. Optical measurements of frequency-dependent linear viscoelastic moduli of complex fluids. Phys. Rev. Lett. 74, 1250–1253 (1995). 10058972 10.1103/PhysRevLett.74.1250

[b27] Mason T. G. Estimating the viscoelastic moduli of complex fluids using the generalized Stokes-Einstein equation. Rheol. Acta 39, 371–378 (2000).

[b28] Dasgupta B. R., Tee S. Y., Crocker J. C., Frisken B. J. & Weitz D. A. Microrheology of polyethylene oxide using diffusing wave spectroscopy and single scattering. Phys. Rev. E 65 10.1103/PhysRevE.65.051505 (2002). 12059562

[b29] Evans R. M. L. Transforming from time to frequency without artefacts. BSR Bulletin 50, 76–86 (2009).

[b30] Tassieri M., Evans R. M. L., Warren R. L., Bailey N. J. & Cooper J. M. Microrheology with optical tweezers: data analysis. New J. Phys. 14, 10.1088/1367-2630/14/11/115032 (2012).

[b31] Zaragoza O. *et al.* Fungal Cell Gigantism during Mammalian Infection. PLoS Pathog. 6, 10.1371/journal.ppat.1000945 (2010). PMC2887474 20585557

[b32] Atakhorrami M. *et al.* Correlated fluctuations of microparticles in viscoelastic solutions: Quantitative measurement of material properties by microrheology in the presence of optical traps. Phys. Rev. E 73, 10.1103/PhysRevE.73.061501 (2006). 16906830

[b33] Preece D. *et al.* Optical tweezers: wideband microrheology. J. Opt. 13, 10.1088/2040-8978/13/4/044022 (2011).

[b34] Tassieri M. *et al.* Measuring storage and loss moduli using optical tweezers: Broadband microrheology. Phys. Rev. E 81, 10.1103/PhysRevE.81.026308 (2010). 20365652

[b35] Pommella A. *et al.* Using Optical Tweezers for the Characterization of Polyelectrolyte Solutions with Very Low Viscoelasticity. Langmuir 29, 9224–9230 (2013). 23786307 10.1021/la4015948 PMC3730292

[b36] Bennett J. S. *et al.* Spatially-resolved rotational microrheology with an optically-trapped sphere. Sci. Rep. 3, 10.1038/srep01759 (2013).

[b37] Berg-Sorensen K. & Flyvbjerg H. Power spectrum analysis for optical tweezers. Rev. Sci. Instrum. 75, 594–612 (2004).

[b38] Munk P., Aminabhavi T. M., Williams P., Hoffman D. E. & Chmelir M. Some solution properties of Polyacrylamide. Macromolecules 13, 871–875 (1980).

[b39] Gibson G. M., Leach J., Keen S., Wright A. J. & Padgett M. J. Measuring the accuracy of particle position and force in optical tweezers using high-speed video microscopy. Opt. Express 16, 14561–14570 (2008). 18794991 10.1364/oe.16.014561

